# Relative Biological Effectiveness (RBE) of ^131^I Radiation Relative to ^60^Co Gamma Rays

**Published:** 2013-08-24

**Authors:** Ali Neshasteh-Riz, Ali Mahmoud Pashazadeh, Seyed Rabie Mahdavi

**Affiliations:** 1 Faculty of Allied Medicine, Department of Radiation Sciences, Tehran University of Medical Sciences ,Tehran, Iran; 2 The Persian Gulf Nuclear Medicine Research Center, Bushehr University of Medical Sciences, Bushehr, Iran

**Keywords:** RBE, Glioblastoma, Spheroid, Photons, Electrons

## Abstract

**Objective:**

To assess relative biological effectiveness (RBE) of ^131^I radiation relative to ^60^Co gamma rays in glioblastoma spheroid cells.

**Materials and Methods:**

: In this experimental study, glioblastoma spheroid cells were exposed to ^131^I radiation and ^60^Co gamma rays. Radiation induced DNA damage was evaluated by alkaline comet assay. Samples of spheroid cells were treated by radiation from ^131^I for four different periods of time to find the dose-response equation. Spheroid cells were also exposed by 200 cGy of ^60^Co gamma rays as reference radiation to induce DNA damage as endpoint.

**Results:**

Resulted RBE of ^131^I radiation relative to ^60^Co gamma rays in 100 µm giloblastoma spheroid cells was equal to 1.16.

**Conclusion:**

The finding of this study suggests that ^131^I photons and electrons can be more effective than ^60^Co gamma rays to produce DNA damage in glioblastoma spheroid cells.

## Introduction

In nuclear medicine, ^131^I is one of the most commonly used radioisotopes in treatment of thyroid. Application of this radioisotope in treatment of thyroid-related diseases has been one of the most successful treatments in radiation therapy. Recently some studies have been performed in order to assess the treatment capability of ^131^I radiation in central nervous system (CNS) tumors. Application of ^131^I labeled with metaiodobenzylguanidine (MIBG) has shown promising therapeutic effects in treatment of neuroblastoma. In one study, performed on 42 patients with advanced neuroblastoma, the overall survival for stage three and stage four were 75 and 69% respectively (1). Post-surgery radioimmunotherapy of patients with glioblastoma has also shown promising results in controlling the progression of the residual tumor. Direct injection of ^131^I labeled antitenascin antibodies into the tumor mass of 30 patients has lead to overall response rate of 34.7% (2). Because of increasing studies in therapeutic application of ^131^I, there is a need to further study the basic radiobiology of this radioisotope. For this reason and in order to establish appropriate protocols in medical application of ^131^I, we measured relative biological effectiveness of ^131^I, as a comprehensive radiobiological concept. Relative biological effectiveness (RBE) is defined as the ratio of a dose of standard radiation to the dose of test radiation to produce the same biological effects. Although 250 kVp X-ray was the common standard radiation to determine the RBE, the International Commission on Radiation Protection (ICRP) recommended in their 92^th^ report to use gamma rays of ^60^Co as reference radiation (3) and therefore was chosen as reference in this study. In spite of the fact that ^60^Co gamma rays and ^131^I radiation as two low linear energy transfer (LET) radiations have the same quality factor (QF), they seem to exert different biological effects. Determination of RBE is a comprehensive way to compare effects of these two low LET radiations. ICRP recommended in 1990 a quality factor of 1 for all low LET radiations such as photons and electrons but* in vitro* studies have shown different biological effects for photons and electrons (4). Although ICRP in 2007 accepted that low LET radiations have different effects on cells but it still continues to use quality factor of 1 for all low LET radiations (5).

Depending on the energy, low LET radiations have different biological effects and different RBEs. In general, low energy radiations are more effective in comparison to high energy radiations. For example, 29 kVp X-ray is more effective than 200-220 kVp X-rays (6) or Tritium beta ray (5.7 keV) is much more effective than 15 MeV electrons (7). In order to compare biological effects of two types of radiation a variety of endpoints can be used. Radiation induced DNA damage to detect the primary effects of radiation on biological cells can be used as the biological endpoint. In addition, there are efficient and robust lab techniques such as comet assay or single cell gel electrophoresis in which extent of DNA damage in cells, so called tail moment, is a measurable quantity and have been used for more than two decades (8, 9).

In this study, in order to understand the basic radiobiology of ^131^I radiation and to compare biological effects of two low LET radiations, we investigated the relative biological effectiveness of ^131^I radiation to ^60^Co gamma rays in spheroids of U87MG cell line using the comet assay.

## Materials and Methods

### Thermoluminescent dosimeter (TLD) calibration

In this experimental study, TLD-100 chips with dimensions of 1.3×1.3×0.9 mm3 , density of 2.64 g/cm3 and average atomic number of 8.2 were used as tissue equivalent dosimeter. Twelve TLD chips were annealed at 400˚C for 1 hour followed by second an-w nealing at 100˚C for 24 hours. For calibration purpose, TLD chips in four groups of three were irradiated by ^60^Co gamma rays of doses 10, 30, 50, 70, 90 and 110 cGy. In order to apply the correction factor in the calibration equation, a second exposure was performed at different doses of 5, 10, 40, 60, 80 and 100 cGy. During irradiation, a tissue equivalent plexiglass layer was covered on TLD chips to provide build up region of ^60^Co photons. Thermo luminescent reading was performed by TLD reader Harshaw/Bicron model 3500.

### Dosimetry of ^131^I radiation by TLD

In order to determine the dose-time equation of ^131^I radiation in TLD chips, each group of TLD chips was covered by a thin layer of plastic and was embedded in a flask. All flasks were filled by 10 ml of medium and 10 mCi of ^131^I. TLD groups were exposed for 30, 60, 90, 120 and 150 minutes respectively

### Cell line

U87MG cell line was obtained from Pastor Institute of Iran. It was cultured in Minimal Essential Medium (MEM) (Gibco, USA) containing 10% Fetal Bovine Serum (FBS) (Gibco, USA) and 500µ/ ml of penicillin (Sigma, USA).

### Monolayer culture

Glioblastoma cells were cultured as a monolayer in T-25 flasks (NUNC, Denmark) under the incubation condition of 37˚C, 5% CO_2_ and humidified atmosphere of 95%. In subculturing process, Phosphate Buffer Saline (PBS) was used for washing cells and 1 mM ethylenediaminetetraacetic acid (EDTA) was used for trypsinizing the cells.

### Spheroid culture

Spheroids were cultured by liquid Overlay technique. Number of 105 cells were cultured in 100 mm dishes that were coated with a layer of 1% agar with 10 ml of MEM containing 10% FBS. The plates were incubated in 5% CO_2_ , 37˚C and humid-h ified atmosphere. Every three days half of medium was removed and replaced with fresh medium.

### Spheroid growth curve

Each spheroid cell was transferred into a multiwell plate (24 wells/plate) (NUNC, Denmark) that was coated by 1% agar with 10ml of MEM supplemented with 10% FBS. The spheroid cells were incubated at 5% CO_2_ , 37˚C. In order to calculate the volume of each spheroid according to the equation V (Volume)=a×b^2^×π/6, two perpendicular diameters of the spheroid (a and b) were measured to find the growth equation V(t)=V_0_×e^kt^, where V_0_ is the initial volume of spheroid and k represents gradient of logarithmic phase of the growth curve

### Cell treatment by ^60^Co radiation

Glioblastoma Spheroid cells were irradiated by photons of ^60^Co as reference radiation. Theratron 780 unit at dose rate of 80 cGy per minute was used to deliver dose of 200 cGy to spheroid cells. Flasks containing spheroid cells were sufficiently filled with medium and covered the cells to provide build up layer of ^60^Co photons. Exposure factors were set at field size of 12×12 cm^2^ and SSD of 70 cm. In order to measure damages based on factors other than ^60^Co radiations, one flask was not exposed as the control group. Resulted damages of unexposed spheroids were used to determine net damages of ^60^Co radiation.

### Cell treatment by ^131^I radiation 

In order to find dose-response equation of ^131^I radiation in glioblastoma cells, spheroids in various culture flasks were exposed for different exposure times (30, 60, 90 and 120 minutes). Initial activity of 10 mCi ^131^I was poured into each flask of spheroids. The control sample was also considered in order to find net damages of ^131^I radiation.

### Viability test 

In order to evaluate viability of cells in each category, a suspension of each category was mixed with Trypan blue. The viability test determines the ability of cells to recover its viability. In each step, before counting the cells under microscope, viability test was performed to determine the health status of cells. The ratio of cell suspension to trypan blue was 9:1. The mixture was observed by a light microscope (Leica, DMLS, USA) and all the blue cells were considered as dead cells. Percentage of unstained cells, as healthy cells, to the total cells represented the viability.

### Comet assay

Induced DNA damage in U87MG cells exposed to ^60^Co radiation and ^131^I radiation were determined by alkaline comet assay. Microscope slides were coated with 1% normal melting point agarose (Merck, Germany). After counting spheroid cells in hemocytometer, approximately 10000 cells in 10 µl PBS were mixed with 100 µl of 5% low melting agarose (Merck, Germany). The cell suspension was then poured on the coated microscope slide. After getting the suspension on the slides solidified, they were immersed in lysis buffer (2.5 M NaCl, 100 mM EDTA, 10 mM Tris-base with 1% Triton X-100, pH=10) and incubated for one hour.

Subsequently, the slides were removed from the buffer and transferred into a denaturation buffer (300 mM NaOH, 1mM EDTA, pH=13) in a horizontal gel electrophoresis tank (Cleaver Scientific Ltd, CSL-COM20). Electrophoresis was performed for 30 minutes at the voltage of 1 V/ cm and amperage of 300 mA. After electrophoresis, alkaline was neutralized by Tris buffer (0.4 M Tris-HCl, pH=7.5) and the slides were then immersed in ethidium bromide. In order to take photograph of damaged cells, slides were transferred under a fluorescent microscope (Zeiss, Axioskop 2 plus) with an ethidium bromide filter (excitation filter, 535 nm; emission filter, 610 nm) and a CCD camera (Hitachi, KP-D20BP).

### Evaluation of DNA damage

For each sample of cells three slides were considered and for each slide 100 cells were scored. DNA damage was quantified as increase in tail moment. The intensity of comet tail relative to the head of comet represented DNA damage. In most experiments, cell damages with respect to the extent of damaged DNA, could be scored from no damage (class one) to total damage (class five). Highly damaged form of DNA may result in apoptosis. DNA tail moments were analyzed by comet score software.

### Determination of RBE 

RBE is the ratio of the dose of reference radiation to a dose of test radiation to produce a similar endpoint. In this experimental study, ^60^Co was used as reference radiation and net induced DNA damage by 200 cGy of this radiation was considered as the endpoint. In order to find dose of ^131^I beta radiation (D_test_) which produces the same endpoint, dose-response equation of ^131^I radiation was determined. In this equation, response was equaled to the induced endpoint (response) by 200 cGy of ^60^Co gamma photons. Finally, ratio of 200 cGy to the determined dose of ^131^I (D_test_) beta radiation was considered as RBE.

## Results

**Fig 1:**
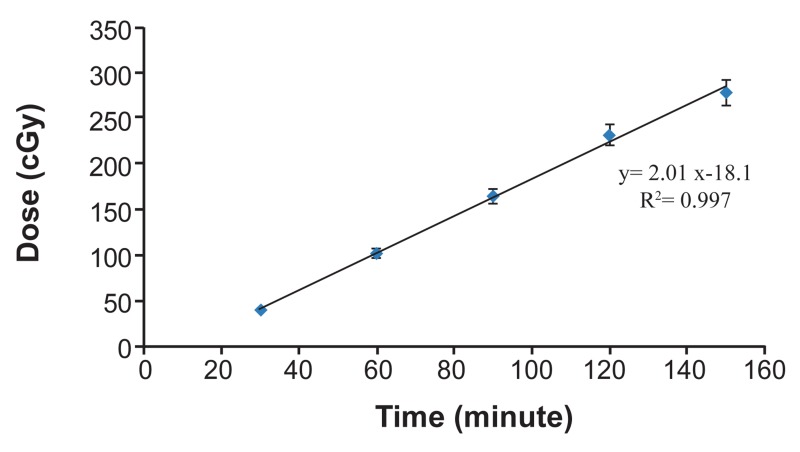
Absorbed doses in TLD chips by irradiation of ^131^I for 30, 60, 90, 120 and 150 minutes. (Error bars represent Mean ± SEM of 3 replicates).

1I and related absorbed dose in TLD chips are represented in table 1. Corresponding time-dose equation is shown in figure 1 indicating linearity for this relationship (R^2^ =0.997). 

**Table 1:** Absorbed doses in TLD chips by ^131^I radiation


Irradiation time (minutes)	Absorbed dose (cGy)

30	40
60	102
90	164
120	231
150	277


**Fig 2:**
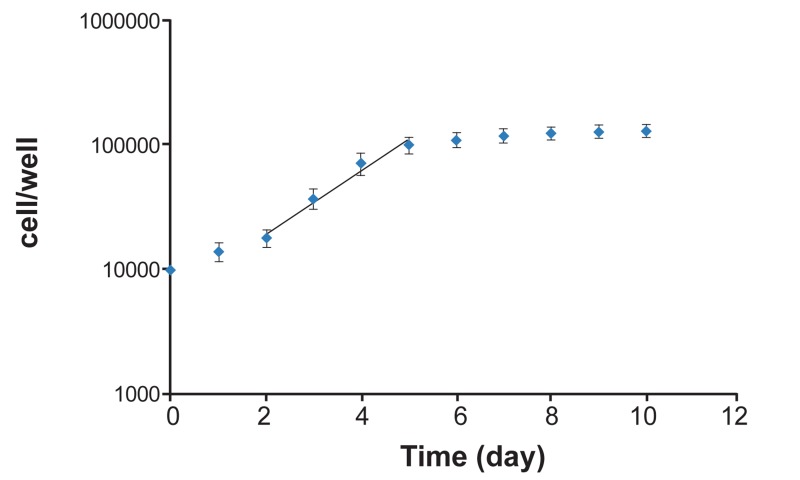
Growth curve of U87MG cell line in the spheroid cultures (y=6999e0.561X). The log phase of curve is in days 2 to 5 and gradient of this phase is used to measure the volume doubling time (calculated doubling time is 67 hours). (Error bars represent Mean ± SEM of 3 replicates).

**Fig 3:**
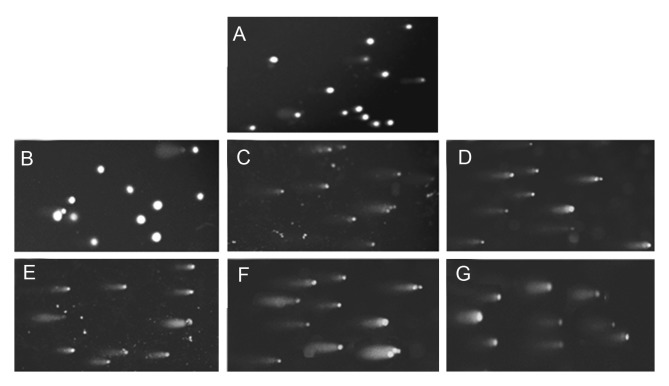
Comet Assay photos of U87MG cells of 100μm spheroids after irradiation with ^131^I and ^60^Co. Microphotographies are representative of the following slides: A. control for ^60^Co, B. control for ^131^I, C. irradiated by ^131^I for 30 minutes, D. irradiated by ^131^I for 60 minutes, E. irradiated by ^131^I for 90 minutes, F. irradiated by ^131^I for 120 minutes, G. irradiated by 200 cGy of ^60^Co.

The U87MG cell line was cultured as spheroid forms. Growth curve of spheroids is shown in figure 2 and this curve was used to determine the doubling time (67 hours). It lasted 11 days to have spheroids with Relative Biological Effectiveness of ^131^I Radiation 100 μm in diameter. After exposure of cells, induced DNA damage in cells was evaluated as tail moments by comet assay. Figures 3A-3G represent microphotographies of the 100 μm spheroid cells in control and treated groups. Apoptosis as a result of highly damaged DNA was also observed. (some DNA damages such as DNA-cross link, DSB and bulky DNA adducts, if not repaired, may lead to apoptosis). Irradiation was performed in two different places for ^131^I and ^60^Co irradiation and due to different environmental conditions, induced base damage in control groups for ^131^I and ^60^Co irradiation were not similar.

**Fig 4:**
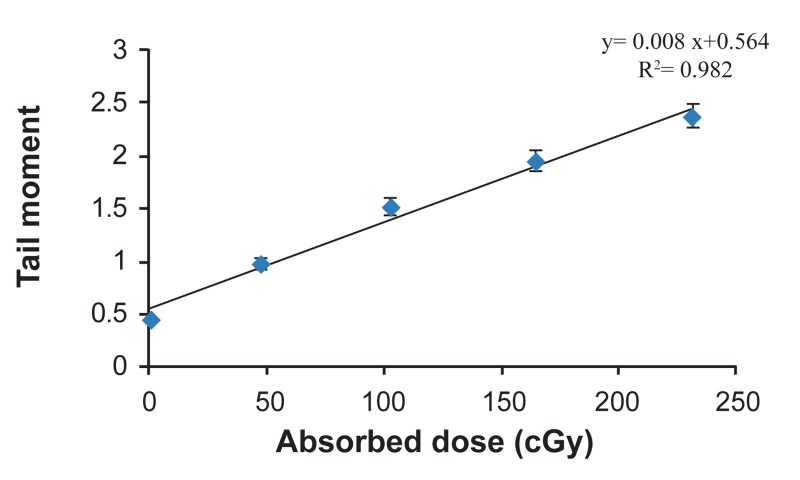
Resulted tail moments in U87MG cell lines of 100μm spheroids relative to absorbed doses of ^131^I irradiation (Error bars represent Mean ± SEM of 3 replicates).

**Fig 5:**
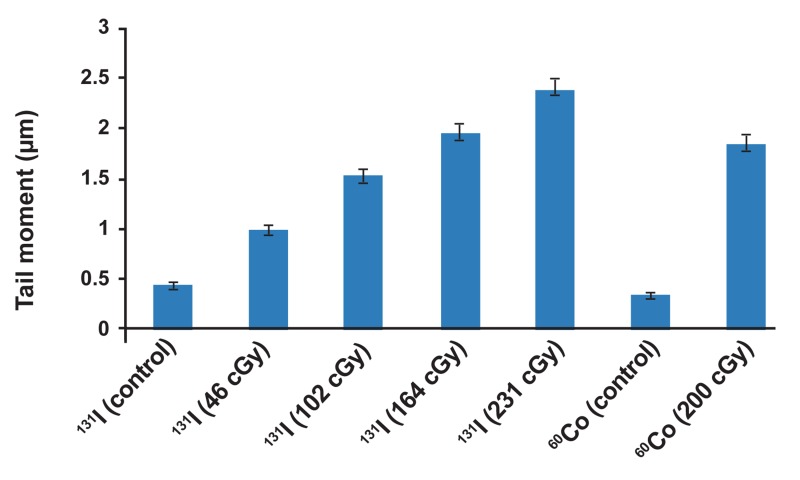
Comparative chart for induced tail moment in irradiated groups by radiations of ^131^I and ^60^Co (Error bars represent SD of 3 replicates).

For each category of cells the average tail moment, was determined as induced DNA damage. Table 2 represents absorbed dose and corresponding DNA damage as tail moments in glioblastoma spheroid cells. Dose-response equation of ^131^I radiation was Y=0.008X+0.564, where X and Y are absorbed dose in cGy and tail moment in µm as response of cells to the radiation respectively (Fig 4). In order to obtain net damage, DNA damage in control group was subtracted from treated groups. Resulted dose-response equation for net damage induced by ^131^I radiation was Y=0.008X+0.116. With respect to this equation, 171.1 cGy of ^131^I radiation and 200 cGy of ^60^Co radiation have the same biological effect. Therefore, ratio of 200 cGy to 171.1 cGy, equal to 1.16, was found to be the RBE of ^131^I radiation to ^60^Co radiation. RBE depends on many factors such as dose, dose rate, cell line and endpoint. RBE in this experiment is resulted from the dose-response equation that showed increasing the exposure time to ^131^I radiation, increases DNA damage in glioblastoma tumor cells. Also the correlation coefficient showed that there is good linearity between absorbed dose of ^131^I in 100μm spheroid cells and resulted DNA damage (R^2^ =0.982). Comparative chart for irradiated groups by radiations of ^131^I and ^60^Co is shown in figure 5.

**Table 2:** Induced tail moments in glioblastoma spheroid cells by ^131^I and ^60^Co radiations


Radioisotope	Absorbed dose (cGy)	Tail moment (μm)

^131^I	0 (control)	0.44
^131^I	46	0.98
^131^I	102	1.52
^131^I	164	1.94
^131^I	231	2.37
^60^Co	0 (control)	0.35
^60^Co	200	1.84


## Discussion

RBE is a complex concept to compare a test radiation with reference radiation in a comprehensive way. RBE depends on dose, dose rate, LET, expose conditions, cell type and endpoint (10) and is used for radiation treatment planning and radiation protection. For a good treatment planning by ion beams, in order to focus maximum RBE to the center of tumor, knowledge about dependence of RBE to depth is necessary. In radiation protection, RBE is a key factor to derive QF (6).

In this study, RBE of ^131^I radiation was evaluated relative to ^60^Co gamma rays in an experimental approach (10). RBE of 1.16 indicated that ^131^I radiation is more effective than ^60^Co radiation to produce DNA damage in 100 µm glioblastoma spheroid cells. According to previous studies, RBE of ^131^I is in the range of 0.3 to 1(11). This variation may be due to factors affecting RBE.

One of the affecting factors on RBE is energy. Experimental studies have shown that low energy photons and electrons are more effective than high energy photons or electrons to produce biological damage (6). For ^60^Co and ^131^I radiation radiobiological mechanisms are different which result in different biological effects. High energy photons of ^60^Co as indirect ionizing radiation produce energetic secondary electrons by Compton scattering and photoelectric interaction whereas ^131^I radiation consists of photons and beta particles. Beta particles of ^131^I have average range of one millimeter therefore increase DNA damage due to the cross fire phenomenon (12, 13). Capability of ^131^I beta particles in producing considerable damages in small clusters of cells has made it a promising radioisotope in target therapy of small tumors. After successful use of ^131^I in thyroid cancer treatments, researchers have focused on treatment of other malignancies such as hepatocellular carcinoma (HCC) and pheochromocytoma (PCC) by radio iodine (14, 15). ^131^I-MIBG (Metaiodobenzylguanidine) is a radio labeled antibody in target therapy of CNS tumors which uses ^131^I to damage tumor cells (16). Since CNS tumors are resistant to radiation and therapies such as chemotherapy and external beam therapy are restricted because of proximity of critical organs to these tumors, target therapy can be an alternative.

Findings of this study can be used in radiation therapy or target therapy of glioblastoma tumors.

It is clear that comparison between biological effects of ^131^I radiation and ^60^Co gamma rays at the DNA scale forms the basis of the information for understanding radiobiology of ^131^I but more comprehensive studies are needed in this field. Type of cell line is also the other factor which affects the RBE. Further studies are recommended to evaluate biological effects of ^131^I radiation on various cell lines. Also, in this study, ^131^I radiation was evaluated at low dose rate relative to ^60^Co gamma rays at high dose rate. Biological effect of a radiation is affected by dose rate where at low dose and low dose rate, cellular repair mechanism operates and reduces biological effects of radiation (6). Therefore, further studies are recommended to assess RBE of ^131^I at various dose rates to find possible relation between dose rate and RBE of ^131^I radiation. Relationship between dose rate and RBE will be important in health risks in which exposure to the radioiodine occurs at various dose rates. Moreover, it is important in radiation therapy to find the optimal dose rate to achieve optimum treatment.

## Conclusion

This study was an attempt to measure the RBE of ^131^I in a new approach with high sensitive techniques at the DNA scale. It is indicated that beta particles of ^131^I, due to cross fire phenomenon, have an important role in inducing considerable damage in small spheroid cells. The result of this study (RBE of 1.16) differs from previous findings, indicating dependency of RBE to the conditions of the experiment.
